# Thermal photodynamic therapy increases apoptosis and reactive oxygen species generation in cutaneous and mucosal squamous cell carcinoma cells

**DOI:** 10.1038/s41598-018-30908-6

**Published:** 2018-08-22

**Authors:** Evan Austin, Eugene Koo, Jared Jagdeo

**Affiliations:** 1 Dermatology Service, Sacramento VA Medical Center, Mather, CA USA; 2 Department of Dermatology, University of California at Davis, Sacramento, CA USA; 3 Department of Dermatology, State University of New York, Downstate Medical Center, Brooklyn, NY USA

## Abstract

Thermal photodynamic therapy (PDT) is an emerging modality to optimize treatment of pre-cancerous squamous cell carcinoma (SCC) lesions, known as actinic keratoses. Thermal PDT involves heating the tissue, skin, or mucosa above normal skin temperature during 5-aminolevulinic (5-ALA) incubation and irradiating with blue light, which leads to cell apoptosis and reactive oxygen species (ROS) generation. To our knowledge, thermal PDT has not been studied for the treatment of cutaneous or mucosal SCC. We incubated two SCC cell lines with 5-ALA for 30 minutes at temperatures between 21 °C and 42 °C and then irradiated cells with 1000 seconds of blue light. We measured changes in apoptosis, necrosis, and ROS. At 36 °C, there was a dose-dependent increase in apoptosis and ROS generation. Thermal incubation of 5-ALA at 39° and 42 °C followed by blue light increased cell apoptosis and ROS generation compared to untreated control samples incubated at the same temperatures. Thermal PDT may represent a new treatment option for cutaneous and mucosal SCC cancer. Thermal PDT is associated with an increase in SCC cellular apoptosis and is associated with an upregulation in ROS. Clinical trials are required to determine optimal thermal PDT treatment parameters and efficacy for cutaneous and mucosal SCC.

## Introduction

Thermal photodynamic therapy (PDT) is an emerging modality designed to optimize treatment of pre-cancerous squamous cell carcinoma (SCC) lesions, known as actinic keratoses (AKs). Classically, PDT is a two-step process in which application of a photosensitizer, such as 5-aminolevulinic acid (5-ALA), is followed by activation of the photosensitizer by visible light irradiation. 5-ALA is typically converted into heme, but cancerous and other aberrant cells lack the enzyme ferrochelatase, which converts the intermediate product, protoporphyrin IX (PP-IX), into heme. Thus tumor cells have increased PP-IX content relative to normal cells^1,2^. Visible light irradiation induces the formation of free radical reactive oxygen species (ROS) by PP-IX excitation. ROS subsequently induces cellular death via apoptotic pathways. During thermal PDT, the tissue, skin, or mucosa is heated above normal skin temperature (33° to 34° C) during 5-ALA incubation, which enhances 5-ALA uptake and PP-IX formation^3,4^.

To our knowledge, thermal PDT has not been studied for the treatment of cutaneous or mucosal SCC. SCC includes malignant transformation of keratinocytes (i.e. cutaneous SCC) or epithelial tissue (i.e. mucosal SCC) including oropharyngeal and vulvar surfaces. European and American guidelines and clinical evidence recommend non-thermal PDT for cutaneous and mucosal SCC *in-situ*^5–10^. Thermal PDT approaches may be more effective in treating aggressive, invasive, or deeply penetrating cutaneous and mucosal SCC lesions. Optimizing PDT with heating of skin or mucous membranes during ALA incubation may be desirable for treatment of cutaneous and mucosal SCC, as PDT is non-invasive, can be topically applied, has a track record of excellent cosmesis, and no reports of scaring^5–10^.

Cutaneous and mucosal SCC is a significant public health issue and new treatments, such as thermal PDT, are needed. Cutaneous SCC is the second most common cancer in Europe and the United States behind basal cell carcinoma (BCC)^11–13^. The annual incidences of cutaneous SCC per 100,000 person-years in England, Scotland, and Northern Ireland are reported to be 22.65, 27, and 30.6, respectively^14^. Globally, there are greater than 2.5 million cases of cutaneous SCC and 13,000 deaths per year as of 2006^15^. Oral SCC is the third most common cancer of the head and neck behind BCC and cutaneous SCC^16^. In the United States, 0.7 to 1.7% percent of female patients have vulvar SCC, and in England, it is estimated that 2.51% of female patients have vulvar cancer, of which SCC is the predominant type^17,18^. Treatments for cutaneous and mucosal SCC include surgical excision, fluorouracil, imiquimod, radiation therapy, and PDT^5,8^. From 2007 to 2011, over 4.5 billion US dollars were spent treating non-melanoma skin cancers (a classification which includes SCC and BCC), a 74% increase in spending from the previous 4-year period^19^. In the Netherlands, the total cost for treatment of recurrent/metastatic SCC of the head and neck was 10,000 to 40,000 Euros with the primary drivers of cost being hospital stays and cancer drugs^20^. It is estimated by 2025, non-melanoma skin cancer will cost the United Kingdom 338 to 465 million pounds per year^21^.

There is a need to further investigate thermal PDT as a potential therapeutic modality for cutaneous and mucosal SCC. We have previously studied thermal PDT in our laboratory as a method to optimize outcomes during therapy using dermal fibroblast cells, and herein, we sought to expand our thermal PDT research by studying effects on cutaneous and mucosal SCC cells^5,7,22–24^. We hypothesized that thermal PDT would be an effective therapeutic modality for cutaneous and mucosal SCC by increasing apoptosis and ROS generation. We thermally incubated cutaneous (SCC-13) and mucosal (A431) SCC cells and measured changes in apoptosis, necrosis, and ROS. We found that thermal PDT is an effective method to augment destruction of cutaneous and mucosal SCC by increasing apoptosis and ROS.

## Results

**Figure 1:**
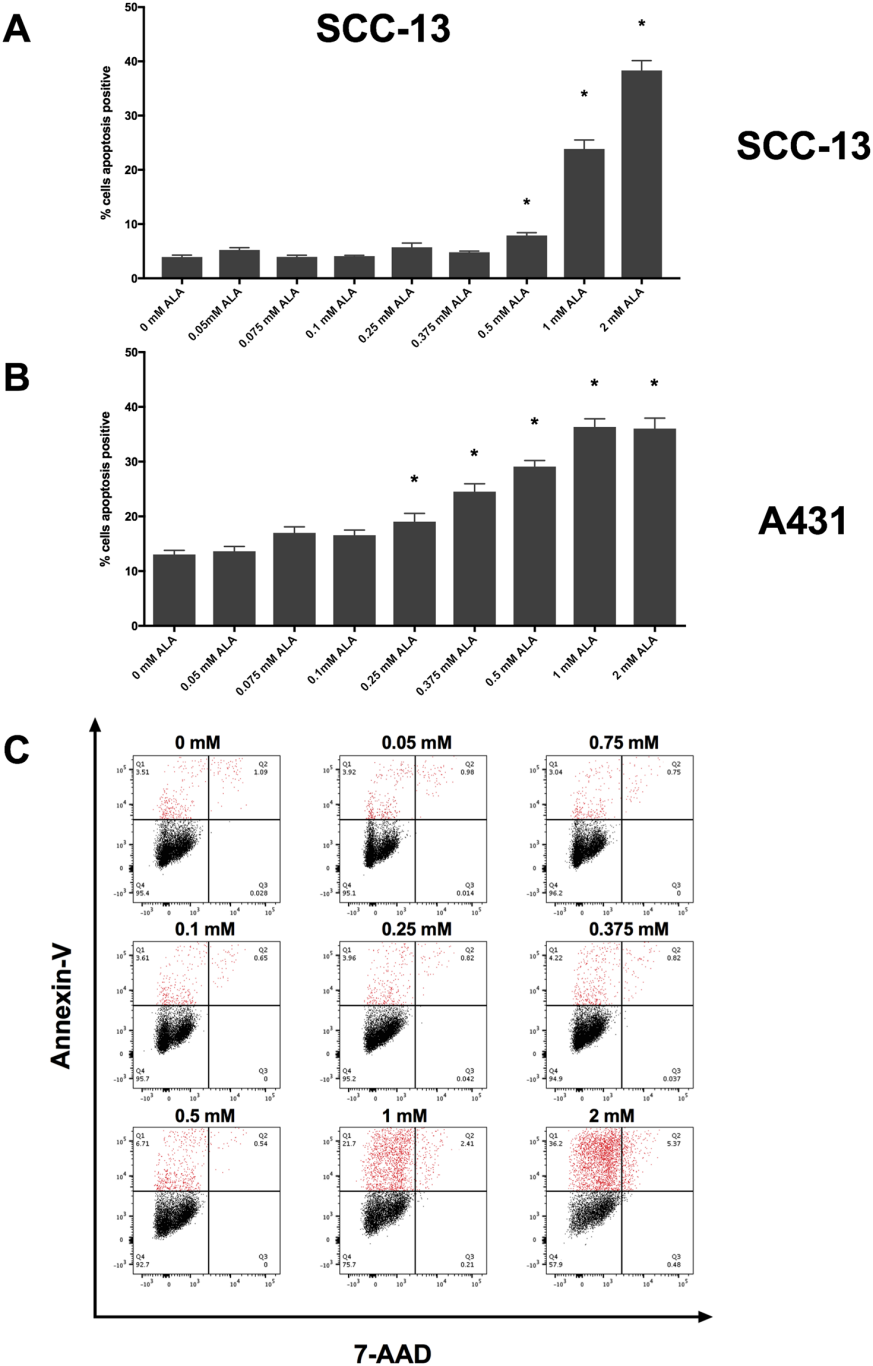
Thirty minutes incubation of 5-ALA induced a dose-dependent increase in apoptosis. (**A**) 5-ALA incubated at 36 °C for thirty minutes followed by 1000 seconds of blue light induced a dose-dependent increase in apoptosis in SCC-13 and (**B**) A431 cells. Bars represent average percent annexin-V positive cells in each treatment and control group. Error bars represent the mean ± standard error of the mean. *P < 0.05. (**C**) Representative annexin-V vs. 7-AAD flow cytometry plots for 0, 0.075, 0.1, 0.25, 0.375, 0.5, 1, and 2 mM 5-ALA incubated cells. Q1 – early apoptotic cells, Q2 – late apoptotic/necrotic cells, Q3 – necrotic cells, Q4 – viable cells. Apoptotic cells populations are highlighted in red. Experiments were performed in technical triplicate.

To determine if SCC-13 and A431 respond to PDT in a dose-dependent manner, we incubated SCC-13 and A431 cells with 0, 0.05, 0.075, 0.1, 0.25, 0.375, 0.5, 1, and 2 mM 5-ALA at 36 °C for 30 minutes and irradiated cells with 1000 seconds of blue light. Immediately following PDT treatment, we measured changes in apoptosis and ROS using annexin-V/7-aminoactinomycin D (7-AAD) and dihydroethidium (DHE) flow cytometry, respectively. In SCC-13 and A431 cell lines, we measured a 5-ALA dose-dependent increase in cell apoptosis (Fig. 1). In SCC-13, there was a significant increase in apoptosis when incubated with 0.5 mM (7.9 ± 0.52%), 1 mM (23.86 ± 1.49%), and 2 mM (38.33 ± 1.81%) 5-ALA (Table 1). In A431 cells, there was a significant increase in cell apoptosis at concentrations of 0.25 mM (19.05 ± 1.51%), 0.375 mM (24.53 ± 1.42%), 0.5 mM (29.1 ± 1.10%), 1 mM (36.33 ± 1.49%), and 2 mM (36.04 ± 1.91%) 5-ALA.

**Table 1:** Percent apoptosis of 5-ALA treated samples at variable concentrations.

Percent Apoptosis									
5-ALA concentration	0 mM	0.05 mM	0.075 mM	0.1 mM	0.25 mM	0.375 mM	0.5 mM	1 mM	2 mM
SSC-13	3.94 ± 0.34	5.24 ± 0.41	3.95 ± 0.32	4.11 ± 0.13	5.72 ± 0.79	4.81 ± 0.21	7.90 ± 0.52*	23.86 ± 1.64*	38.33 ± 1.81*
A431	13.04 ± 0.75	13.63 ± 0.88	16.98 ± 1.11	16.55 ± 0.96	19.05 ± 1.51*	24.53 ± 1.42*	29.10 ± 1.10*	36.33 ± 1.49*	36.04 ± 1.91*

Abbreviations: 5-ALA – 5-aminolevulinic acid. P < 0.05 denoted by asterisk (*).

**Figure 2:**
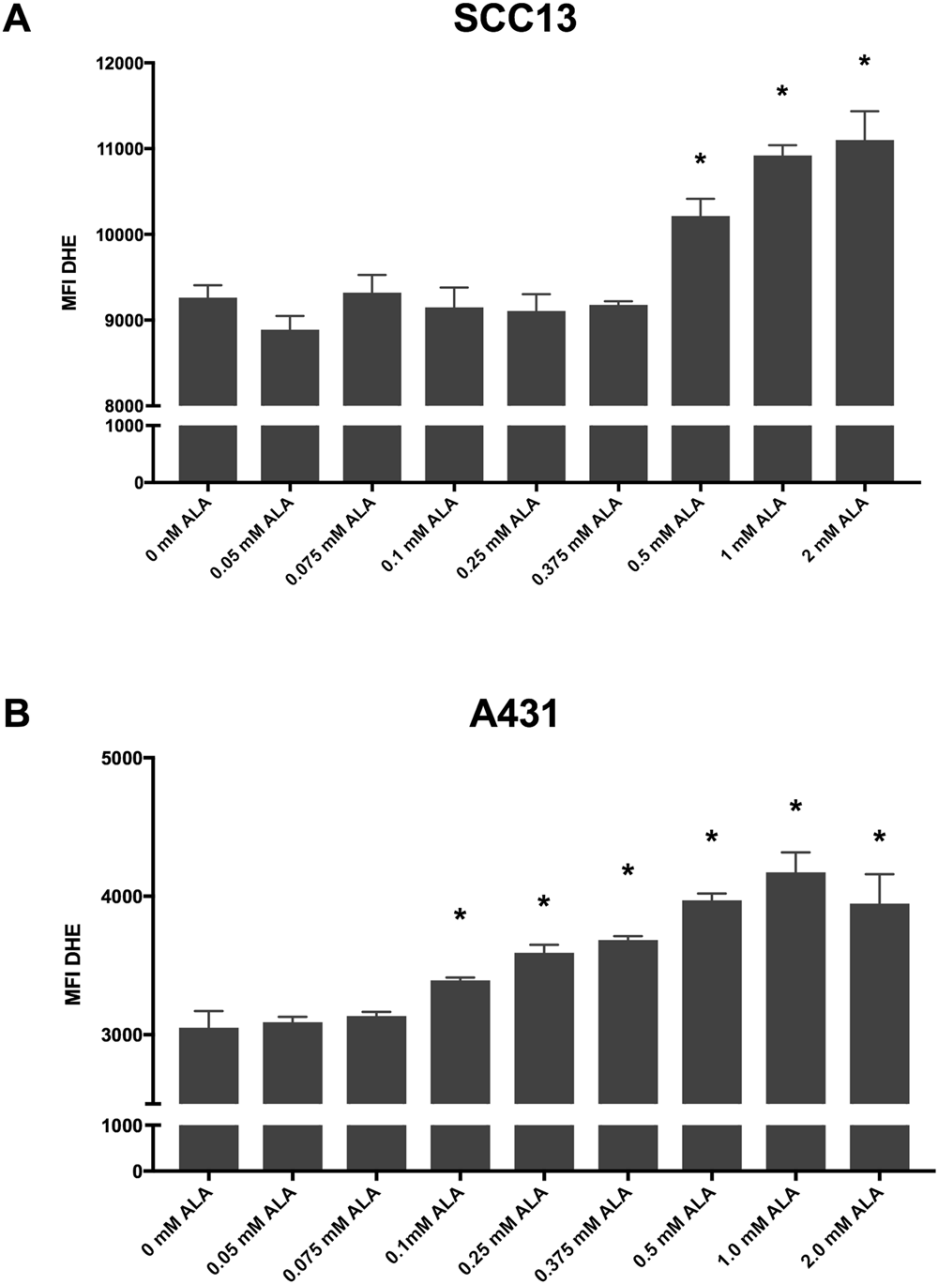
Thirty minutes incubation of 5-ALA induced increased ROS generation in a dose-dependent manner. (**A**) SCC-13 and (**B**) A431 cells incubated with 5-ALA at 36° for thirty minutes followed by 1000 seconds of blue lights induced a dose-dependent increase in ROS generation. Error bars represent mean ± standard error of the mean; *P < 0.05 compared to 0 mM control.

ROS generation was similarly increased in SCC13 and A431 cells (Table 2). In SCC-13 cells, 0.5 mM, 1 mM, and 2 mM 5-ALA led to a dose-dependent increase in ROS generation (Fig. 2A). In A431, there was a similar significant increase in ROS at concentrations of 0.1 mM to 2 mM 5-ALA compared to 0 mM 5-ALA (Fig. 2B).

**Table 2:** ROS generation of 5-ALA treated samples at variable concentrations.

ROS (MFI)									
5-ALA concentration	0 mM	0.05 mM	0.075 mM	0.1 mM	0.25 mM	0.375 mM	0.5 mM	1 mM	2 mM
SSC-13	9261.67 ± 144.84	8888.67 ± 160.13	9320.00 ± 207.23	9149.00 ± 230.79	9106.33 ± 196.61	9177.00 ± 42.6	10,213.67 ± 201.07*	10,921.00 ± 117.97*	11,09.67 ± 335.72*
A431	3051.33 ± 120.35	3091.33 ± 37.55	3134.67 ± 29.72	3393.00 ± 20.84*	3591.00 ± 58.11*	3732.67 ± 74.20*	3921.00 ± 96.89*	4173.00 ± 144.31*	3947.33 ± 212.57*

Abbreviations: 5-ALA – 5-aminolevulinic acid, ROS – Reactive Oxygen Species, MFI – Median Fluorescent Intensity. P < 0.05 denoted by asterisk (*).

**Figure 3:**
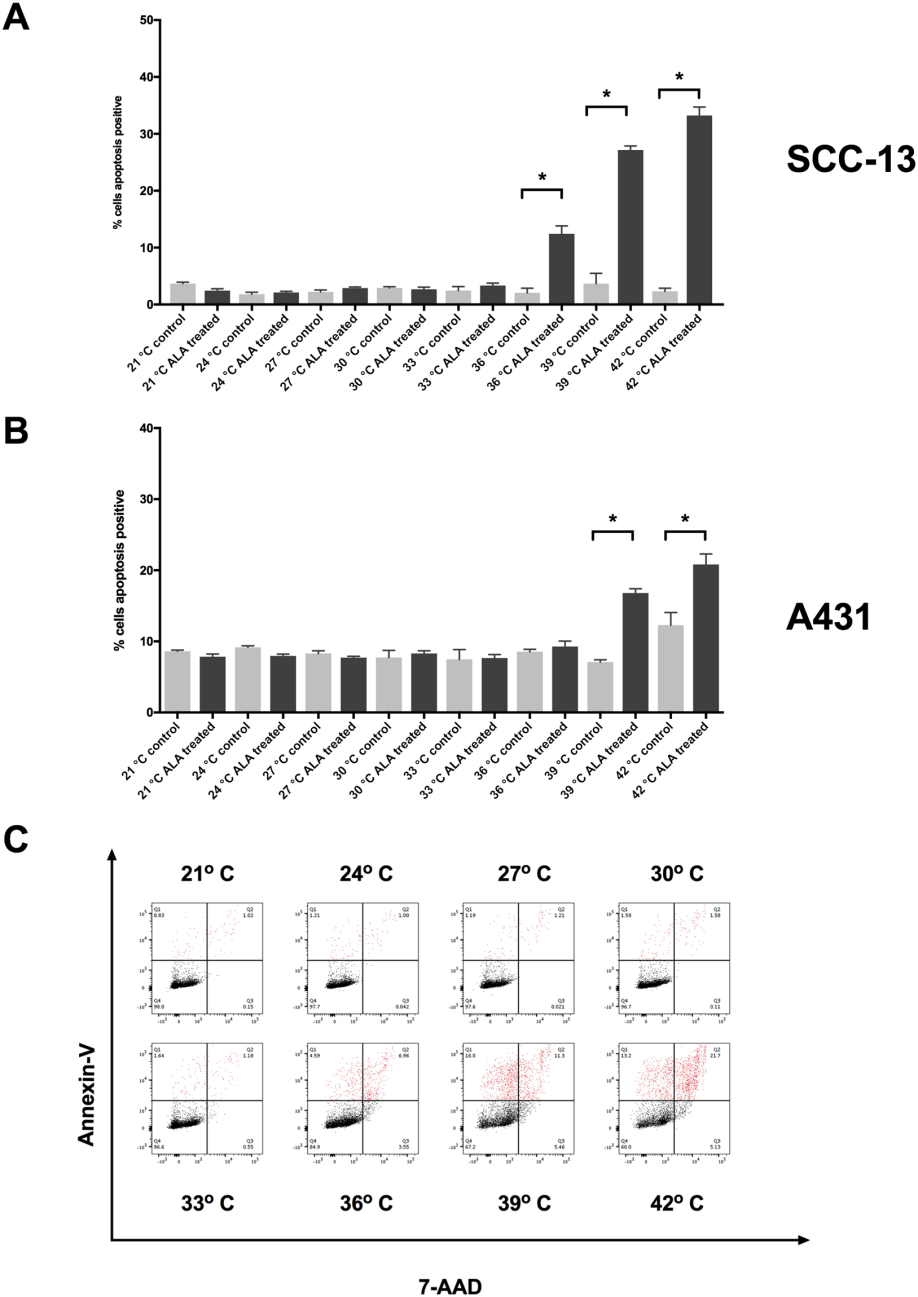
Thermal 5-ALA incubation induced apoptosis in SCC-13 and A431 cells. (**A**) 5-ALA incubated at 21 °C to 42 °C for thirty minutes followed by 1000 seconds of blue light induced a temperature-dependent increase in apoptosis in SCC-13 and (**B**) A431 cells. Bars represent average percent annexin-v positive cells in each treatment and control group. Error bars represent the mean ± standard error of the mean. *P < 0.05. (**C**) Representative annexin-V vs. 7-AAD flow cytometry plots for 21, 24, 27, 30, 33, 36, 39, 42 °C thermal incubation. Q1 – early apoptotic cells, Q2 – late apoptotic/necrotic cells, Q3 – necrotic cells, Q4 – viable cells. Apoptotic cells populations are highlighted in red. Experiments were performed in triplicate.

**Figure 4:**
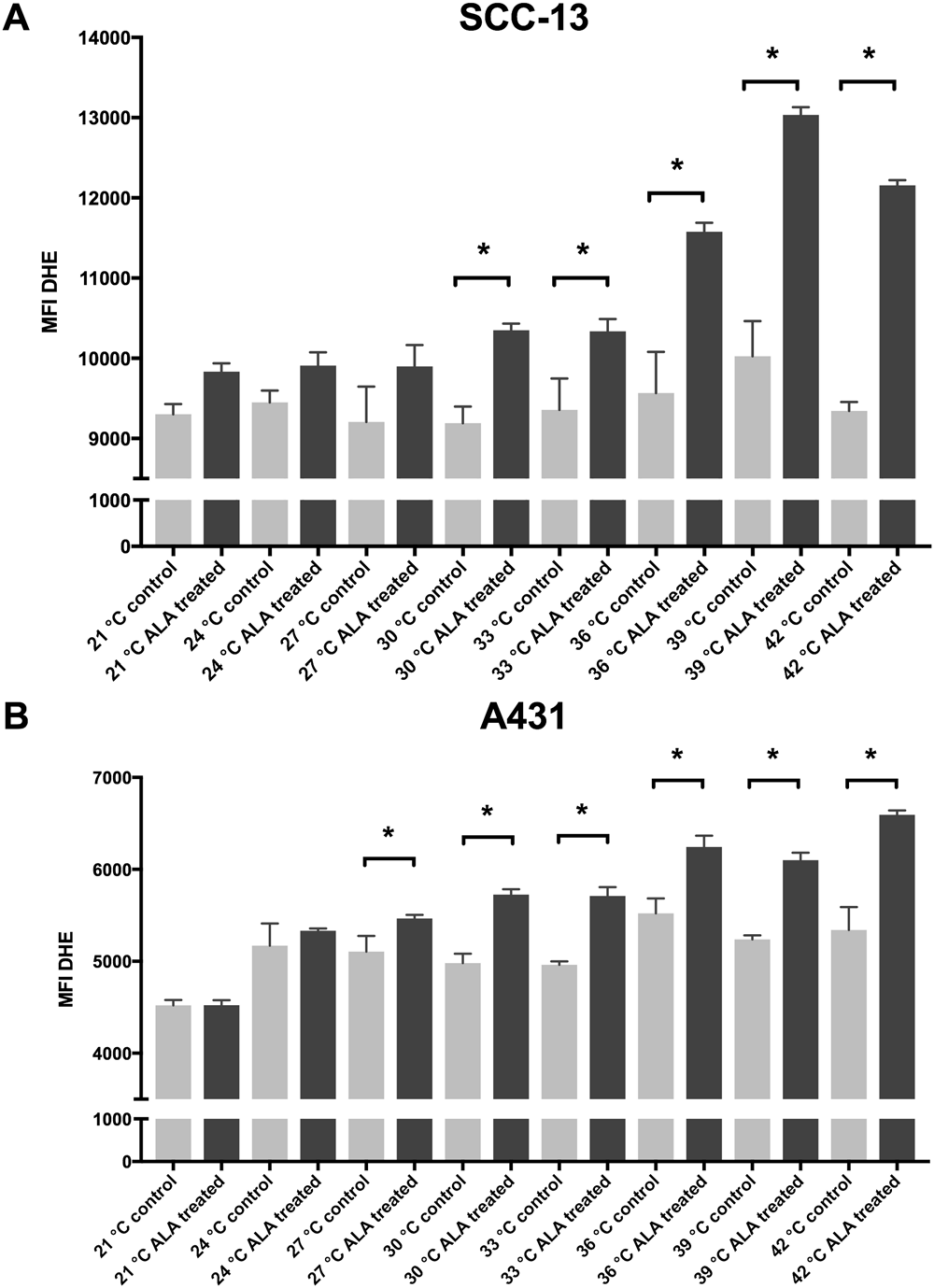
Thermal incubation of 5-ALA increased ROS generation in A431 and SCC-13 cells. (**A**) SCC-13 and (**B**) A431 cells thermally incubated with 5-ALA for thirty minutes followed by 1000 seconds of blue lights increased ROS generation. Error bars represent mean ± standard error of the mean; *P < 0.05 compared to 0 mM control.

Next, we sought to determine whether thermal incubation of 5-ALA enhances the efficacy of PDT. We incubated SCC-13 and A431 cells with 0.5 mM 5-ALA (the inflection point from aforementioned dosing experiments) and measured changes in apoptosis and ROS generation compared to temperature matched control samples. In both SCC-13 and A431 cells, we observed a temperature-dependent increase in apoptosis following thermal incubation (Fig. 3). At temperatures of 36°, 39°, and 42 °C, there was 12.43 ± 1.39, 27.15 ± 0.71, and 33.23 ± 1.49% apoptosis in SCC-13 cells, respectively (Table 3). Thermal incubation in A431 cells resulted in 16.80 ± 0.60 and 20.82 ± 1.48% apoptosis at 39° and 42 °C, respectively. ROS generation following thermal incubation was similarly increased (Table 4). At temperatures of 30° to 42 °C in SCC-13 cells and 27° to 42 °C in A431 cells, there was a significant increase in ROS compared to non-5-ALA treated control samples (Fig. 4).

**Table 3:** Percent apoptosis of 5-ALA and control samples incubated at variable temperatures.

Percent Apoptosis								
Temperature	21 °C	24 °C	27 °C	30 °C	33 °C	36 °C	39 °C	42 °C
SSC-13								
5-ALA	2.45 ± 0.33	2.11 ± 0.21	2.89 ± 0.65	2.69 ± 0.36	3.34 ± 0.87	12.43 ± 1.39*	27.15 ± 0.71*	33.23 ± 1.49*
Control	3.67 ± 0.25	1.80 ± 0.21	2.24 ± 0.19	2.92 ± 0.13	2.46 ± 0.40	2.03 ± 0.13	3.66 ± 1.06	2.30 ± 0.32
A431								
5-ALA	7.85 ± 0.38	7.96 ± 0.24	7.71 ± 0.20	8.30 ± 0.37	7.66 ± 0.49	9.28 ± 0.75	16.80 ± 0.60*	20.82 ± 1.48*
Control	8.60 ± 0.18	9.16 ± 0.13	8.30 ± 0.21	7.74 ± 0.58	7.46 ± 0.80	8.54 ± 0.19	7.09 ± 0.19	12.29 ± 1.02

Abbreviations: 5-ALA – 5-aminolevulinic acid. P < 0.05 denoted by asterisk. (*).

**Table 4:** ROS generation of 5-ALA and control samples incubated at variable temperatures.

ROS (MFI)								
Temperature	21 °C	24 °C	27 °C	30 °C	33 °C	36 °C	39 °C	42 °C
**SSC-13**								
5-ALA	9833.33 ± 103.19	9909.67 ± 163.98	9899.00 ± 266.70	10,349.67 ± 81.43*	10,335.67 ± 152.43*	11,578.67 ± 110.92*	13,034.33 ± 96.06*	12,156.67 ± 64.09*
Control	9301.67 ± 73.09	9449.33 ± 85.53	9205.00 ± 255.34	9192.00 ± 119.46	9355.67 ± 226.82	9565.67 ± 296.33	10,025.33 ± 253.61	9343.33 ± 63.83
**A431**								
5-ALA	4522.33 ± 55.41	5332.00 ± 24.70	5466.00 ± 39.00*	5726.00 ± 58.23*	5710.33 ± 96.20*	6242.67 ± 124.71*	6099.33 ± 81.49*	6593.00 ± 48.68*
Control	4521.67 ± 32.77	5169.33 ± 139.26	5105.67 ± 98.20	4980.33 ± 59.48	4962.00 ± 21.13	5522.33 ± 25.67	5238.67 ± 35.67	5340.33 ± 143.87

Abbreviations: 5-ALA – 5-aminolevulinic acid, ROS – Reactive Oxygen Species, MFI – Median Fluorescent Intensity. P < 0.05 denoted by asterisk (*).

## Discussion

To determine the effect of thermal PDT on cutaneous and mucosal SCC cells, we treated SCC-13 and A431 cells with thermal PDT and measured changes in apoptosis/necrosis and ROS generation. At 36 °C, there was a dose-dependent increase in apoptosis and ROS generation in both cell lines indicating that ultra-short (30 minutes) incubations of 5-ALA followed by 1000 seconds of blue light PDT induced cell death. Thermal incubation of 0.5 mM 5-ALA at 39° and 42 °C followed by blue light increased cell apoptosis and ROS generation compared to 5-ALA untreated temperature-matched control samples incubated at the same temperatures. As a result, we determined that increases in apoptosis and ROS were due to thermal PDT and not associated solely with heat. ROS generation, but not apoptosis, increased when SCC-13 and A431 cells were incubated at 27°, 30°, and 33 °C. Given that it is well established that increases in ROS are mechanistically linked to PDT induced cell death, we extrapolate that thermal PDT increases cell apoptosis by increasing ROS generation^2^. A minimum threshold of ROS and PP-IX generation may be necessary following thermal incubation of 5-ALA before significant apoptosis is induced^25^.

To our knowledge, no other researchers have examined the effects of thermal PDT in cutaneous or mucosal SCC in clinical studies or a laboratory environment. Previously we demonstrated that thermal 5-ALA incubation of 15 minutes and longer may increase cell apoptosis and necrosis in human fibroblast cells at temperatures of 42 °C and below^22^. In previously published research we found that a thirty-minute incubation at 42 °C in human dermal fibroblasts resulted in a similar increase in apoptosis^22^. A431 cells had a smaller but significant increase in apoptosis likely due to a higher baseline apoptotic rate. Differences in baseline apoptotic rates among cell lines may be due to inherent differences in cell turnover, culture medium, and sensitivity to ROS. Changes in ROS were modest in SCC-13 and A431 compared to previously published human dermal fibroblast cells^22^. However, cancer cells have been to shown to have a dysregulated cell redox state compared to normal cells^26^. Thus, the observed modest increases in ROS in a cancer cell may reflect an increased antioxidant state. Furthermore, cancer cells may have a lower tolerance to suprathreshold ROS^26^. As cancer cells are more susceptible to suprathreshold changes in ROS, thermal PDT may overwhelm the redox balance leading to increased cell death^26^. 42 °C is an important temperature threshold as higher temperatures are often reported as uncomfortable or painful, and temperatures at or below are reported by patients as warm or hot.

Other researchers have studied thermal PDT in laboratory keratinocyte and fibroblast cultures, animals and patients. In HaCaT keratinocyte cells, incubation with 2 mM 5-ALA for 3 hours at temperatures of 20°–50 °C followed by blue light PDT resulted in a temperature-dependent increase in cell apoptosis, necrosis, 5-ALA uptake, and PP-IX formation^3^. However, above 44 °C, apoptosis was thermally induced in HaCaT cells indicating a risk for thermolytic effects to healthy tissue^22^. Mouse skin heated to 42 °C and incubated with 5-ALA for 10 minutes resulted in significantly increased PP-IX production compared to normal skin temperature^27^. In clinical practice, 1 hour of 5-ALA PDT at a maximum temperature of 41.2 °C resulted in 90% clearance of slightly thickened (i.e. grade I) and moderately thickened (i.e. grade II) AK lesions at 3 and 12-month follow-up in 17 patients^4,28^. Hypertrophic (i.e. grade III) AKs did not respond to thermal PDT, but the investigators did not debride the AKs^4,27^. This may explain the lower efficacy of thermal PDT in their trial for hypertrophic (grade III) lesions as physically debriding grade III AKs may enhance 5-ALA penetration and efficacy^4,28,4,28^.

As thermal PDT is non-invasive, patients may undergo multiple treatments sessions to target multifocal lesions with relatively low-risk for adverse events. 5-ALA is applied to an entire anatomic area, covering lesional and non-lesional skin. There is no direct contraindication for PDT on any anatomical location and PDT is often performed on facial, extremity, and genital regions. In previous studies, application of a heating pack or space warmer to upper and lower extremities during the incubation phase of thermal PDT was well-tolerated by patients^4^. Therefore, thermal incubation of 5-ALA is likely safe for other anatomical locations. After 5-ALA incubation, the field is irradiated with light to treat the entire anatomical area, accounting for multifocal lesions.

This study has several strengths that enhance the relevance of our study. These include using 5-ALA and a commercially available blue light source with irradiation parameters that match clinical treatment recommendations. We performed ultra-short and thermal PDT experiments on cutaneous (SCC-13) and mucosal (A431) SCC cells, that expand on previous research by our group and other researchers focused on the effects and mechanisms of thermal PDT in non-malignant fibroblasts and keratinocytes^3,22,24,29^. Our *in vitro* model of thermal PDT has a few limitations. SCC-13 and A431 cells were directly exposed to 5-ALA solutions in an adherent cell culture model. The 5-ALA concentrations used to induce apoptosis *in vitro* may not directly correspond to clinical practice. In clinical applications, hyperkeratosis from SCC cancer cells may limit 5-ALA cellular absorption. Debriding SCC lesions before 5-ALA application may enhance absorption and 5-ALA depth of penetration. Additionally, current research has the examined the use of novel nano-particle vehicles for 5-ALA that may increase 5-ALA tissue penetration compared to an alcohol vehicle^30^. Furthermore, we assessed thermal PDT in SCC13 and A431 cells after a single treatment session of 5-ALA incubated for 30 minutes, but cutaneous and mucosal SCC may require longer 5-ALA incubation periods and multiple treatment sessions to yield satisfactory patient outcomes. In clinical practice, 5-ALA is commonly non-thermally incubated on the skin for 1 to 2 hours^6^. As SCC recurrence is a current limitation of classic PDT, other researchers have studied PDT efficacy and mechanism in resistant SCC-13 cells, which have undergone 10 cycles of PDT^31^. In future studies, we may assess the effects of thermal PDT in resistant SCC-13 cells to determine whether thermal PDT can render these cells susceptible to increased rates of cell death.

In conclusion, we found that thermal PDT induced cell death and ROS generation in cutaneous and mucosal SCC cells. Therefore, thermal PDT may represent a new treatment option for cutaneous and mucosal SCC. Clinical trials are required to determine optimal thermal PDT treatment parameters and efficacy for cutaneous and mucosal SCC.

## Methods

### Cell Culture

Mucosal A431 SCC cells (ATCC; Manassas, VA) were cultured in 1 g/L glucose Dulbecco’s Modified Eagle’s Medium (Gibco; Carlsbad, CA) with 10% fetal bovine serum (Atlanta Biologics; Atlanta, GA) and 1% antibiotic-antimycotic (Gibco) mixture. Cutaneous SCC-13 cells (a generous gift from Dr. Carolyn Lee; originally cultured by Dr. Jim Rheinwald) were cultured in keratinocyte serum-free medium (Gibco) supplemented with 100 ng epidermal growth factor and 12.5 mg total bovine pituitary extract^32^. The cell cultures were maintained in a humidified incubator at 37 °C with 5% carbon dioxide. Cells were plated at 20,000 cells per well in a 6-well dish (Corning; Corning, NY).

### 5-ALA Thermal Incubations

Twenty-four hours following plating, SCC-13 and A431 cells were treated with 0.05 to 2 mM 5-ALA (DUSA pharmaceuticals; Wilmington, MA) and incubated for thirty minutes on a custom-designed heating block (Torrey Pines Scientific; Torrey Pines, CA) at 36 °C to determine dose response^24^. We also incubated SCC-13 and A431 cells with 0.5 mM 5-ALA at temperatures of 21° to 42°C to determine the effects of thermal incubation, as previously described^24^.

### Irradiation

SCC-13 and A431 cells were irradiated with blue light (Blu-U; Dusa Pharmaceuticals) for 1000 seconds, as previously described^24^. Briefly, cells were washed and replenished with culture medium following 5-ALA treatment. The cells were placed under a uniform field of blue light (420 ± 5-nm) on a black surface. The blue light was measured to have a power density of approximately 10 W/cm^2^ at 5 cm. After 1000 seconds of blue light, the total fluence was calculated to be 10 J/cm^2^.

### Flow Cytometry

Immediately following blue light irradiations, cells were collected and flow cytometry was performed using a FACSAria cytometer (BD; Franklin Lakes, NJ). Flow cytometry allows for single-cell analysis of physical characteristics of cells. The analysis was performed using FlowJo software (Ashland, OR), a software package used for flow cytometry gating and analysis. ROS generation was quantified by the median fluorescent intensity of the total singlet cell population. For apoptosis, quartile gating of annexin-V and 7-AAD subpopulations were based on a 70 °C heat-treated control (data not shown).

### Apoptosis/Necrosis

We assessed apoptosis and necrosis using annexin-V/7-AAD flow cytometry, as previously described^24^. Immediately following blue light irradiation, the cells were washed, trypsinized, collected, and stained with 1:40 annexin–V (CF-647; Millipore Sigma; St. Louis, MO) to annexin flow buffer for 15 minutes. Annexin-V binds to phosphatidylserine, a membrane protein that is normally oriented towards the intracellular domain. During early apoptosis, phosphatidylserine flips towards the extracellular domain and allows for annexin-V binding. After annexin-V staining, we added 3 µl of 7-AAD per 200 µl of cell suspension and incubated for 5 minutes at room temperature. 7-AAD intercalates nucleic acids but is cell membrane impermeable. During late apoptosis, the integrity of the cell and nuclear membrane is compromised, which allows for 7-AAD binding to the nucleus in addition to annexin-V binding, leading to fluorescence. Cells were analyzed by flow cytometry to determine the percentage of cells that were positive for annexin-V and/or 7-AAD. Early apoptosis is defined by high fluorescence intensity of annexin-V and low fluorescence intensity of 7-AAD. Late apoptosis is defined by high fluorescence intensity of both 7-AAD and annexin-V.

### Free Radical Reactive Oxygen Species Generation

A431 and SCC-13 cells were assayed using dihydroethidium (Sigma) flow cytometry as previously described^24^. DHE specifically measures generation of superoxide free radical ROS, which has been mechanistically linked as the ROS moiety involved in PDT cell death^2^. Briefly, cells were pre-stained with 30 µg/ml DHE in culture medium before blue light irradiation. Cells were detached, collected, and analyzed by flow cytometry.

### Statistics

We compared the median fluorescent intensity of DHE and percent apoptotic cells using the Student’s t-test and analysis of variance with Tukey’s or Dunnett’s multiple comparison testing using GraphPad Prism software (La Jolla, Ca). Experiments were performed in technical triplicate. A P-value of less than 0.05 was considered statistically significant.
